# Medical Decision-Making Incapacity among Newly Diagnosed Older Patients with Hematological Malignancy Receiving First Line Chemotherapy: A Cross-Sectional Study of Patients and Physicians

**DOI:** 10.1371/journal.pone.0136163

**Published:** 2015-08-21

**Authors:** Koji Sugano, Toru Okuyama, Shinsuke Iida, Hirokazu Komatsu, Takashi Ishida, Shigeru Kusumoto, Megumi Uchida, Tomohiro Nakaguchi, Yosuke Kubota, Yoshinori Ito, Kazuhisa Takahashi, Tatsuo Akechi

**Affiliations:** 1 Department of Psychiatry and Cognitive-Behavioral Medicine, Nagoya City University Graduate School of Medical Sciences, Nagoya, Japan; 2 Division of Psycho-oncology and Palliative Care, Nagoya City University Hospital, Nagoya, Japan; 3 Division of Respiratory Medicine, Juntendo University Faculty of Medicine and Graduate School of Medicine, Tokyo, Japan; 4 Department of Hematology and Oncology, Nagoya City University Graduate School of Medical Sciences, Nagoya, Japan; The University of Hong Kong, HONG KONG

## Abstract

**Background:**

Decision-making capacity to provide informed consent regarding treatment is essential among cancer patients. The purpose of this study was to identify the frequency of decision-making incapacity among newly diagnosed older patients with hematological malignancy receiving first-line chemotherapy, to examine factors associated with incapacity and assess physicians’ perceptions of patients’ decision-making incapacity.

**Methods:**

Consecutive patients aged 65 years or over with a primary diagnosis of malignant lymphoma or multiple myeloma were recruited. Decision-making capacity was assessed using the Structured Interview for Competency and Incompetency Assessment Testing and Ranking Inventory-Revised (SICIATRI-R). Cognitive impairment, depressive condition and other possible associated factors were also evaluated.

**Results:**

Among 139 eligible patients registered for this study, 114 completed the survey. Of these, 28 (25%, 95% confidence interval [CI]: 17%-32%) were judged as having some extent of decision-making incompetency according to SICIATRI-R. Higher levels of cognitive impairment and increasing age were significantly associated with decision-making incapacity. Physicians experienced difficulty performing competency assessment (Cohen’s kappa -0.54).

**Conclusions:**

Decision-making incapacity was found to be a common and under-recognized problem in older patients with cancer. Age and assessment of cognitive impairment may provide the opportunity to find patients that are at a high risk of showing decision-making incapacity.

## Introduction

Autonomy, defined as an individual’s right to self-determination, is one of the four basic principles of medical ethics [1]. In the field of health care, a patient’s autonomy is exercised through the process of providing informed consent prior to any medical procedures. Sufficient patient decision-making capacity is an essential component of obtaining appropriate informed consent. Making decisions about cancer treatment requires sufficient cognitive capacity to undertake several difficult tasks. Patients have to understand the extent and life-threatening nature of their disease and the likely efficacy and possible adverse effects of different treatment options. Armed with this information, patients need to make a choice about their treatment, taking their priorities in life (e.g. survival vs. quality of life considerations) into account.

In general, decision-making capacity in regard to treatment requires that the patient is able to meet the following four criteria: to understand the relevant medical information, to appreciate the medical consequences of the situation, to make considered treatment choices, and to communicate the choice [2]. A recent systematic review of studies reported that 26% (95% CI: 18%-35%) of inpatients with medical illnesses, excluding only those with severe mental illnesses, had medical decision-making incapacity [3]. Studies focusing on patient decision-making capacity have typically included small heterogeneous disease populations, and some used non-validated measurement methods to assess decision-making capacity of patients [3]. Furthermore, few studies have systematically investigated decision-making incapacity in patients with cancer.

Despite the high rates of incapacity for medical decision-making among hospital inpatients [3], physicians often encounter difficulty in the evaluation of such incapacity [4]. There is a need to explore whether there are patient demographic and clinical characteristics associated with incapacity to assist clinicians to identify which patients may be more likely to exhibit decision-making incapacity.

Cancer is a common disease among older adults [5]. Cognitive impairment [6] and depression [7] are also highly prevalent among older adults. Both of these factors may hamper decision-making capacity [8, 9]. Aging is among the most important risk factors for cognitive impairment, and over 24 million persons who are 60 years or older are estimated to suffer from dementia, the number expected to exceed 42 million in 2020 [10]. A previous systematic review identified some studies reporting an association between decision-making incapacity and cognitive impairment [3]. Approximately 11 percent of adult cancer patients may have clinical depression [11]. Previous research has reported that cancer patients with depression are less likely to agree to undergo aggressive treatment [12]. Despite the accumulating findings, little research has been carried out to comprehensively explore the factors underlying decision-making incapacity among cancer patients.

Several instruments were suggested to assess competency in medical patients [3], although there is no widely recognized gold standard. We previously indicated that Structured Interview for Competency and Incompetency Assessment Testing and Ranking Inventory (SICIATRI) is useful for assessing competency in patients with cancer [13]. Based on this experience, we conducted this observational survey in which patients’ decision-making capacity, as well as a broad range of possibly associated factors were evaluated using validated measures. The purpose of this study was to investigate, among older patients with hematological malignancy receiving first line chemotherapy, the frequency and factors associated with decision-making incapacity, as well as the accuracy of physicians’ recognition of patients’ decision-making incapacity.

## Patients and Methods

### Ethics Statement

This study was approved by the Institutional Review Board and Ethics Committee of Nagoya City University Graduate School of Medical Sciences, Japan, and was conducted in accordance with the principles laid down in the Helsinki Declaration.

### Subjects

The study subjects were older patients with hematological malignancy admitted to the Nagoya City University Hospital, Japan, for inpatient treatment. Eligibility criteria: newly histopathologically diagnosed malignant lymphoma or multiple myeloma; 65 years of age or older; informed about the cancer diagnosis. Exclusion criteria: patients with severe mental or cognitive disorders diagnosed clinically; inability to understand the Japanese language; judged by the treating physician to be unsuitable to participate. Written consent was obtained from each eligible patient after a thorough explanation of the purpose and method of the study. When the participants could not understand the contents of the study protocol fully, both the patients’ oral consent and surrogates’ written consent were obtained.

### Procedures

Consecutive patients were recruited upon admission to the hospital prior to meeting with their primary physician to discuss the potential benefits and adverse effects of chemotherapy. By means of fixed questionnaire, the investigators then performed the assessments described below, either prior to, or within the first week of, first-line chemotherapy.

### Measurements

#### Decision-making capacity

The Structured Interview for Competency Incompetency Assessment Testing and Ranking Inventory-Revised (SICIATRI-R) consists of 12 items [14, 15]: 1) Is aware that he/she was informed; 2) Understands that he/she has a right to decide; 3) Evidences his/her choice; 4) Does not waive responsibility; 5) Understands the expected benefits; 6) Understands the expected risks; 7) Understands the alternative treatments; 8) Understands the risks expected from no treatment; 9) Understands the benefits expected from no treatment; 10) Wants to get better; 11) Psychological determinants do not exist; 12) Insight. A psychiatrist asked the patients each of the questions and scored the responses, and the patients were finally rated as one of five different competency levels: Levels 0 (entirely incompetent) to 4 (fully competent). The validity and reliability of the SICIATRI have been established in Japanese oncology settings [16]. We utilized a modified version of this method, consistent with our previous study [13].

Inter-rater reliability between the independent evaluations of the two psychiatrists was rigorously examined in a subsample of 23 consecutive patients. The inter-class correlation coefficient was found to be sufficient (0.72, *P* < .01).

The investigators made sure that all participants received the same explanation about chemotherapy, including discussion of expected risks or benefits, adverse effects, alternative treatments and risks expected from no treatment. The contents were checked using the Disclosure Content Check List (DCCL) [14].

#### Potentially associated variables

Physical domain: Attending physicians assessed the Eastern Cooperative Oncology Group performance status for each patient [17].

Psychological and cognitive domain: The Patient Health Questionnaire 9 (PHQ-9) was administered to assess patient’s depression [18]; the validity of the Japanese version has been verified [19]. This measure consists of nine items, with the same four response options for each item: 0 (not at all) to 3 (nearly everyday). A higher total score indicate more severe depression. The Mini-Mental State Examination (MMSE) [20] was used to evaluate the current severity of cognitive impairment of the patients. In many of the previous studies exploring the association between decision-making capacity and cognitive impairment, cognitive impairment was assessed using the MMSE. The Japanese version of the MMSE has been validated [21]. All patients completed the MMSE. The MMSE is scored from 0 to 30 (better score). For the analyses, the PHQ-9 and MMSE total scores were handled as continuous independent variables.

Medical domain: Information on the diagnosis and stage of cancer was obtained from the attending physicians.

Demographic domain: Information on the age, sex, education, job and household size was obtained from patients.

#### Physicians’ recognition of the patients’ decision-making capacity

Primary physicians, blinded to the results of SICIATRI-R, were orally interviewed about their patients’ decision-making capacity by the research team using the same questions for each patient. Interviews were conducted after the patient completed the SICIATRI-R on the same day.

### Statistical Analysis

We estimated the frequency of incompetency with 95% CIs for patients who were interviewed using the SICIATRI-R.

Univariate analysis was carried out to explore which variables were associated with incapacity (with participants dichotomized based on having a SICIATRI-R score of 4 [fully competent] vs. score of 0–3 [some extent of incompetency]; Table 1). Inter-group differences in categorical, non-parametric and continuous variables were tested by the chi-square test (or Fisher’s exact test), Wilcoxon’s rank sum test and unpaired *t*-test, respectively. Factors with a *P* < 0.10 were included in the multiple logistic regression analysis with forced entry.

**Table 1:** Distribution of The Level of Incapacity by SICIATRI-R (*n* = 114).

		95%CI	
Level of competency^a^	*n* (%)	Lower	Higher
L0-L3 (incompetent)	28 (24.6)	17	32
L0	7 (6.1)	2	11
L1	6 (5.3)	1	9
L2	1 (0.9)	-1	3
L3	14 (12.3)	6	18
L4 (competent)	86 (75.4)	68	83

^a^Level 0 (L0) of the most impaired status as opposed to Level 4 (L4) of full competence.

Agreement between the results of the SICIATRI-R and attending physicians’ perceptions of patient incapacity was assessed using Cohen’s kappa coefficients. This measures the agreement, taking into account the agreement occurring by chance, and ranges from -1 to 1. A value of zero would indicate accidental agreement. A greater value indicates higher agreement. One traditional interpretation guideline is as follows: No agreement < 0, Slight agreement = 0.01 to 0.20, Fair agreement = 0.21 to 0.40, Moderate agreement = 0.41 to 0.60, Substantial agreement = 0.61 to 0.80, Almost perfect agreement = 0.81 to 1.00, although there are no universally accepted guidelines to interpretations as to what is a good level of agreement [22].

A significance level of *p* < 0.05 was adopted for all statistical analyses, and *P* values reported were 2-tailed. All analyses were conducted using SPSS software, version 20.0J, for Windows (SPSS Inc., 2010).

## Results

### Demographic and clinical characteristics

A total of 173 potential participants were identified for this study. The research team excluded fourteen patients due to severe cognitive impairment and sixteen due to being too physically ill. Of the remaining 139 eligible patients, informed consent for study participation was obtained from 114 (consent rate 82%). Twelve patients refused to questionnaire or just this research, and four were excluded by logistic reason. A further eight patients were excluded because the SICIATRI-R could not be administered within seven days of treatment commencement due to logistic reasons. One patient did not complete the survey.

Table 2 shows the demographic and clinical characteristics of the remaining 114 study participants. The mean (±SD) and median age of the study population were 73.9 (±5.7) and 74 years, respectively. Seventy-one percent of the subjects had malignant lymphoma. Eleven percent of patients had performance status of 3 or 4.

**Table 2:** Demographical and Clinical Characteristics of Patients (*n* = 114).

Sample characteristic		*n*	(%)
Age (years)	mean: 73.9±5.7 (range; 65–90), median: 74		
Sex	Male	64	55.7
Education	High school or higher	68	59.1
Marital status	Married	82	71.3
Household size	Living with someone	99	86.1
Job	Employed (full-time /part-time)	27	23.5
Diagnosis			
Malignant lymphoma		81	71.1
	Hodgkin	1	1.2
	Non-Hodgkin	80	98.8
	Ann Arbor staging		
	I	14	17.2
	II	16	19.8
	III	20	24.7
	IV	31	38.3
	International Prognosis Index 3 or greater	39	48.1
Multiple Myeloma		33	28.9
	International Staging System		
	I	3	9.1
	II	17	51.5
	III	13	39.4
Performance status	0	32	28.1
	1	51	44.7
	2	18	15.8
	3	12	10.5
	4	1	0.9

### Frequency of incapacity

Table 1 presents the distribution of patients across SICIATRI-R levels. Of the 114 patients who completed the SICIATRI-R, 28 (25%, 95% CI: 17%-32%) patients were judged to be incompetent to some extent.

### Factors associated with incompetency: univariate analysis

Table 3 shows univariate analyses of variables potentially associated with competency. Compared to participants who were competent, patients judged to be incompetent were more likely to be older, and to have more severe cognitive impairment and lower education level.

**Table 3:** Factors associated with incompetency: univariate analysis (*n* = 114).

		Competent		Incompetent		
Domain		(*n* = 86)		(*n* = 28)		*p* value
		Mean	S.D.	Mean	S.D.	
Age		73.1	5.6	76.6	5.5	< .01
Performance status		1.1	0.9	1.3	1.1	.28
Cognitive impairment^a^		26.2	2.7	23.7	4.1	< .01
Depression^b^		6.0	4.9	8.0	6.9	.10
		*n*	(%)	*n*	(%)	
Sex	Male	47	54.7	17	60.7	.66
Diagnosis	Malignant lymphoma	61	70.9	20	71.4	1.0
Education	High school or higher	56	65.1	12	42.9	.05
Household size	Living with someone	76	88.4	22	78.6	.22

^a^Cognitive impairment was assessed by the Mini-Mental State Examination.

^b^Depression was evaluated by the Patient Health Questionnaire 9.

### Factors associated with incompetency: logistic regression analysis

Table 4 shows the multiple logistic regression analysis. Older patients and those with more severe cognitive impairment (i.e. lower MMSE score) had higher odds of being classified as incompetent according to the SICIATRI-R.

**Table 4:** Factors associated with incompetency: logistic regression analysis (*n* = 114).

					95% CI	
Independent Variable	Beta	SE	*p* value	Adjusted OR	Lower	Higher
Age	.92	.04	.03	1.10	1.01	1.19
Cognitive impairment^a^	-.18	.08	.02	0.84	0.73	0.97
Education^b^	.65	.49	.19	1.91	0.73	5.01

^a^Cognitive impairment was assessed by the Mini-Mental State Examination

^b^Coded as: 0 = less than high school; 1 = high school or higher.

### Physicians’ recognition of patient incompetency

Total three patients (3%, 95% CI: 0%-6%) were judged to be incompetent by physicians and these three patients were also considered to be incompetent by the SICIATRI-R. Cohen’s kappa was -0.54, indicating that agreement was no greater than what would be expected by chance.

## Discussion

Our results indicated that one quarter of this patient population had some level of decision-making incapacity, although this was rarely detected by the attending physicians. Cognitive impairment and older age were independently associated with incapacity in this study. Level of education was found to be significant at univariate analysis, but did not reach statistical significance at multivariate analysis.

This study was novel in that it focused on patients with cancer aged 65 years and the survey was conducted at the timing that a patient treatment decision-making capacity was required. Also there are several other strengths of this study. The evaluation of patient competency was performed using validated structured interviews. Inter-rater reliability was also confirmed in the study. Patients were consecutively recruited and the rate of refusal was within acceptable limits. Furthermore, we investigated depression and cognitive function by international validated tool as potential factors associated with patient incapacity.

The frequency of incapacity among older patients with cancer in this study was almost the same as past findings of 26% frequency in general medical populations [3]. Our results indicated that medical decision-making incapacity is a significant problem in elderly patients with cancer, although elderly patients with cancer are thought to be more independent than those without neoplastic conditions [23].

No standardized strategy is available to identify patients who may be at increased risk of decision-making incapacity. Our results indicated the assessment of cognitive impairment plays an important role in identifying decision-making incapacity, consistent with previous studies [3]. Further research is required to clarify which specific cognitive functions are related to decision-making capacity. The association between older age and incompetency, independent from cognitive impairment, may be due to sensory decline, low medical literacy, or passive communication style, all of which have been linked to aging [24–26]. These are communication-related factors that are potentially modifiable by using appropriate interpersonal approaches and resources to ensure adequate communication during both the treatment discussion and the capacity assessment. These problems would be overcome according to correspondence. Exploring the factors underlying this association could deepen the understanding of how medical decision-making capacity may be linked to the aging process.

We also found that physicians have difficulty with evaluating patient competency levels, and are more likely to overestimate the patients' competency, consistent with a previous study [27]. One of the reasons for physician overestimation of patient capacity may be that patients who were judged to be incompetent in this study did not refuse the anti-cancer treatment suggested by physicians. Although refusal of treatment does not necessarily indicate incapacity, such expression may alert physicians to re-assess decision-making capacity more carefully. Another reason may be that the presence of family members during the informed consent process may mask the patients’ incapacity, because family members often play key roles in the medical decision-making in Japan.

Recently, implementation of Comprehensive Geriatric Assessment (CGA) in the cancer setting has been recommended. CGA is defined as a multidimensional, often interdisciplinary, diagnostic process aimed at determining the medical, psychological and functional capabilities of older persons in order to develop an overall treatment and long-term follow-up plan [28]. Assessment of cognitive impairment is thought to be an essential component of CGA [29]. Thus, CGA provides the opportunity to find the patients at high risk of incapacity. Furthermore, we suggest integrating assessment of medical decision-making capacity in the CGA.

Several limitations of this study should be acknowledged. Firstly, the sample size was small, and limited to hematological cancer patients. Caution should be exercised when generalizing the results to other populations, such as patients with solid tumor or those younger than 65 years of age. Second, not adopting probability sampling methods entails that study sample may not represent the entire population. But consecutive sampling is considered as the best of all non-probability samples. Third, as this study was conducted at a single tertiary institute, multicenter investigations are warranted. Finally, we did not explore the specific influence of communication on decision-making incapacity.

## Conclusion

Decision-making capacity remains difficult for clinicians to assess. Given the high frequency of incompetency in older adults, and the potential impact that this may have on the patients’ ability to make informed treatment decisions, we need to better understand and identify this problem. This study provides preliminary evidence that age and assessment of cognitive impairment using SICIATRI-R could identify patients with decision-making incapacity, which remained under recognized in older patients with cancer. To maximize patient autonomy, further research is required to develop an appropriate brief assessment or screening tool for physicians to evaluate medical decision-making capacity.
